# A Review of free fatty acid-induced cell signaling, angiopoietin-like protein 4, and skeletal muscle differentiation

**DOI:** 10.3389/fphys.2022.987977

**Published:** 2022-09-06

**Authors:** Yura Son, Chad M. Paton

**Affiliations:** ^1^ Department Nutritional Sciences, Athens, GA, United States; ^2^ Department of Food Science and Technology, University of Georgia, Athens, GA, United States

**Keywords:** lipid metabolism (fatty acids, myogenesis, ANGPTL4, angiopoietin-like 4, differentiation

## Abstract

Postnatal skeletal muscle differentiation from quiescent satellite cells is a highly regulated process, although our understanding of the contribution of nutritional factors in myogenesis is limited. Free fatty acids (FFAs) are known to cause detrimental effects to differentiated skeletal muscle cells by increasing oxidative stress which leads to muscle wasting and insulin resistance in skeletal muscle. In addition, FFAs are thought to act as inhibitors of skeletal muscle differentiation. However, the precise molecular mechanisms underlying the effects of FFAs on skeletal muscle differentiation remains to be elucidated. There is a clear relationship between dietary FFAs and their ability to suppress myogenesis and we propose the hypothesis that the FFA-mediated increase in angiopoietin-like protein 4 (ANGPTL4) may play a role in the inhibition of differentiation. This review discusses the role of FFAs in skeletal muscle differentiation to-date and proposes potential mechanisms of FFA-induced ANGPTL4 mediated inhibition of skeletal muscle differentiation.

## Introduction

Skeletal muscle comprises approximately 40% of body mass and is the largest metabolically active tissue in the human body (Huard et al., 2002). It is developed through the process of myogenesis, which is composed of several different stages including determination, differentiation, and maturation. Among the stages, differentiation is tightly regulated, which results in the formation of myotubes. (Yun and Wold, 1996; Ostrovidov et al., 2014; Chal and Pourquié, 2017). Thus, skeletal muscle differentiation is a pivotal process in embryonic development as well as regeneration and repair throughout the life span (Endo, 2015). There are many factors that affect skeletal muscle differentiation such as growth hormones or chemical compounds including resveratrol and caffeic acid (Kaminski et al., 2012; Maioli et al., 2012; Ye et al., 2013; Jang et al., 2018). Even though skeletal muscle differentiation is a life-long event, there are very few studies that have investigated nutritional effects on skeletal muscle differentiation (Hurley et al., 2006; Magee et al., 2008; Peng et al., 2012).

Of the relatively few studies that have assessed the role of macronutrients on myogenesis, there appears to be consensus on the role of free fatty acids (FFAs) in skeletal muscle physiology and increasing public health concern regarding the effects of a high-fat diet on skeletal muscle physiology (Son and Paton, 2020). For example, FFAs may cause atrophy, myopathy, and insulin resistance in skeletal muscle (Capllonch et al., 2012; Lipina and Hundal, 2017; Pinho et al., 2017; Pennisi et al., 2018). In addition, several FFAs such as palmitate and linoleate may cause detrimental effects on skeletal muscle differentiation (Hurley et al., 2006; Yang et al., 2013). However, little is known regarding the mechanisms of FFA-mediated skeletal muscle differentiation. Therefore, it is important to elucidate the inhibitory mechanisms of FFAs in skeletal muscle differentiation to better understand the biological and physiological impacts of dietary fats on tissue development and subsequent metabolic function. In addition, understanding the impact of high-fat diets on skeletal muscle differentiation could provide clinical evidence to improve the quality of life at various stages of development from birth to late-life.

A potential mechanistic link between dietary fat intake and impaired myogenesis is through FFA-induced expression of angiopoietin like protein 4 (ANGPTL4). ANGPTL4 is a secreted glycoprotein expressed in skeletal muscle and it regulates lipid metabolism by inhibiting lipoprotein lipase activity (Dijk and Kersten, 2014). ANGPTL4 also participates in various non-metabolic processes in the human body such as angiogenesis and tumorigenesis (Köster et al., 2005; Van der Kolk et al., 2016; La Paglia et al., 2017; Carbone et al., 2018). In addition to those functions, this review provides evidence for a potential mechanism by which FFA-mediated impairments of skeletal muscle differentiation that is associated with ANGPTL4 production. In the present review, we discuss the overview of skeletal muscle development, the relationship between FFAs and skeletal muscle differentiation, the effects of FFAs on ANGPTL4 in skeletal muscle, and finally, the potential mechanisms of FFA-induced ANGPTL4-mediated inhibition of skeletal muscle differentiation.

## The effect of obesity on skeletal muscle development

Skeletal muscle development can be affected by genetic as well as environmental factors, such as high-fat diet induced obesity (Fu et al., 2016). A high-fat diet changes biological function in various tissues including skeletal muscle by increasing lipid deposition and metabolic remodeling away from catabolism and toward anabolism of lipid droplets and lipotoxicity (Schrauwen, 2007; Capllonch et al., 2012; Pinho et al., 2017; Ferretti et al., 2018). Lipid accumulation in skeletal muscle affects metabolism, mitochondrial function, insulin signaling, and skeletal muscle maintenance and regeneration, suggesting that obesity and/or a high-fat diet could impair skeletal muscle physiology and possibly myogenesis (Sparks et al., 2005; Akhmedov and Berdeaux, 2013; Miotto et al., 2018).

There are several studies that show an association between obesity and impaired myogenesis, however, the mechanisms governing the impact of a high-fat diet on skeletal muscle cell differentiation remains to be examined. Skeletal muscle progenitor cell function has been shown to be impaired in obese populations and obesity was associated with reduced skeletal muscle differentiation (Akhmedov and Berdeaux, 2013). Fu et al. demonstrated that obese mice had impaired satellite cell activation, which eventually decreased skeletal muscle differentiation and thus skeletal muscle regeneration (Fu et al., 2016). Satellite cells isolated from skeletal muscle in obese older individuals (BMI>25, age 69.5 ± 1.8 years) had impaired myogenesis compared to non-obese population (BMI< 25, age 68.1 ± 3.3 years) (O’Leary et al., 2018).

In addition, maternal obesity affects fetal skeletal muscle development (Tong et al., 2009; Du et al., 2010). Maternal obesity results in a shift of the commitment of mesenchymal stem cells (MSCs) from myogenesis to adipogenesis and fibrogenesis during fetal development which reduces skeletal muscle fiber numbers and size but increases intramuscular fat and connective tissue (Du et al., 2010). Maternal obesity reduced fetal skeletal muscle development by decreasing the expression of MRFs including Pax7, MyoD, and MyoG and by suppressing Wnt/β-catenin signaling in the fetal skeletal muscle (Tong et al., 2009). In light of these findings, it is suggested that a high-fat diet might inhibit skeletal muscle differentiation. However, the mechanism underlying a high-fat diet-induced inhibition of skeletal muscle differentiation is still not fully understood.

## Effects of free fatty acids on skeletal muscle differentiation

FFAs perform various functions in skeletal muscle, including signal transduction, transcriptional regulation, and energy storage/production (Son and Paton, 2020). Despite their essential roles, FFAs can induce various unfavorable health conditions including inflammation, metabolic syndrome, and insulin resistance (Unger, 2003; Dubé et al., 2014). In skeletal muscle, FFAs, especially when provided in excess, have a deleterious influence such as impairing lipid metabolism and mitochondrial function and causing skeletal muscle wasting and myosteatosis (Collino et al., 2014; Ewaschuk et al., 2014; Almasud et al., 2017). Myosteatosis can cause insulin resistance and type 2 diabetes, and it is also associated with cancer (Addison et al., 2014). Excess fat deposition into skeletal muscle results in the reduction of skeletal muscle strength and contractility with increased metabolic abnormalities predominantly attributed to type 2 diabetes, insulin resistance, and impaired glucose disposal.

The inhibitory effects of FFAs on skeletal muscle differentiation have been reported (Table 1), but the mechanism(s) by which they suppress differentiation is still unclear. C2C12 murine myoblasts cells treated with omega-3 polyunsaturated fatty acids (docosahexaenoic acid (22:6n3) + eicosapentaenoic acid (20:5n3)) had fewer myotubes formed during differentiation compared to non-treated cells (Hsueh et al., 2018). Furthermore, omega-3 fatty acid treatment decreased mitochondrial biogenesis and gene expression of Pax7, MyoD, MyoG, and Mrf4. Yang et al. also found that palmitate (16:0) significantly decreased the number of myotubes by ∼50% in C2C12 cells (Yang et al., 2013). Their study found that palmitate but not oleate (18:1n9) decreased the expression of genes associated with skeletal muscle differentiation and myofiber composition (MyoG, MHC1, MHC2b, and MCK) in C2C12 cells. In addition, palmitate significantly decreased myotube size in C2C12 cells when treated for 48 h (by 25%) and 96 h (over 90%) (Bryner et al., 2012). Even though it has been shown to have various health benefits including anti-carcinogenic, anti-inflammatory, anti-obesity, *trans-*10, *cis-*12 conjugated linoleic acid decreased the expression of MyoG (64%), MCK (59%), MHC1 (62%), and MHC2b (85%) genes in C2C12 cells (Hommelberg et al., 2010). Additionally, linoleate (18:2n6) inhibited differentiation at 100–200 μM in L6 myoblast cells, showing decreased creatine kinase content throughout differentiation even though it slightly stimulated differentiation at a lower concentration (12.5 μM) (Hurley et al., 2006). Lastly, Zhang et al. reported that pre-treatment of FFAs including palmitate (16:0), oleate (18:1n9), and linoleate (18:2n6) inhibited skeletal muscle differentiation in both C2C12 and L6 skeletal muscle cells, decreases myotube formation, and MCK levels (Zhang et al., 2012). It is widely known that FFA composition plays a critical role in the cellular response to FFAs, however it may be possible that the energy content of FFAs may be detrimental to myogenesis by promoting excess lipid accumulation. This latter fact has not been tested independent of FFA composition, therefore a better understanding of whether or not composition vs reduced carbon content are separate or additive factors affecting myogenesis should be assessed.

**TABLE 1:** A summary of studies investigated the inhibitory effects of FFAs on skeletal muscle development.

References	Treatment	Outcome	Model
Hsueh et al. (2018)	EPA + DHA	• Myotube formation	C2C12 cells
		• Mitochondrial biogenesis	
		• Gene expression of Pax7, MyoD, MyoG, Mrf4	
Yang et al. (2013)	Palmitate	• Myotube formation	C2C12 cells
		• Gene expression of MyoG, MHC1, MHC2b, MCK	
Bryner et al. (2012)	Palmitate	• Myotube size	C2C12 cells
Hommelberg et al. (2010)	*trans-*10, *cis-*12 conjugated linoleic acid	• Gene expression of MyoG, MHC1, MHC2b, MCK	C2C12 cells
Hurley et al. (2006)	Linoleic acid	• MCK contents	L6 cells
Zhang et al. (2012)	Palmitate, Oleate, Linoleic acid	• Myotube formation	C2C12 cells/L6 cells
		• MCK contents	

EPA, eicosapentaenoic acid; DHA, docosahexaenoic acid; Pax7, paired box gene 7; MyoD, myoblast determination protein 1; MyoG, myogenin; MHC1, myosin heavy chain1; MHC2b, myosin heavy chain 2b; MCK, muscle creatine kinase.

## Angiopoietin-like protein 4 (ANGPTL4)

ANGPTL4 is a secreted glycosylated protein that belongs to the ANGPTL family, and it is the most investigated member of the ANGPTL protein family as a signal protein expressed in high metabolic tissues including skeletal muscle, adipose tissue and the liver (Staiger et al., 2009; Dijk and Kersten, 2014; La Paglia et al., 2017). It is secreted into the circulation and prevents lipoprotein lipase (LPL) dimerization, thereby inhibiting LPL activation (Sukonina et al., 2006). ANGPTL4 also participates in various metabolic processes in the human body such as angiogenesis and tumorigenesis (Köster et al., 2005; Van der Kolk et al., 2016; La Paglia et al., 2017; Carbone et al., 2018). ANGPTL4 is a multifunctional protein, regulating lipid metabolism, food intake, inflammation, angiogenesis, and glucose homeostasis (Grootaert et al., 2012). It has been shown that fasting and a high-fat diet increase ANGPTL4, also known as a fasting-induced adipose factor (FIAF) in plasma (Yang et al., 2018; Kaviani et al., 2019).

**FIGURE 1:**
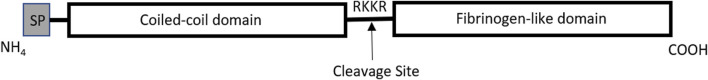
Graphic illustration of ANGPTL4 protein domains. The amino terminal region contains a signal peptide (SP) that is removed during posttranslational processing, leaving the coiled-coil and fibrinogen-like domains. Prior to release from intracellular secretory vesicles, ANGPTL4 is cleaved at the RKKR site, creating the NH_4_- and COOH-terminal fragments of ANGPTL4. Illustration adapted from Köster et. al (Köster et al., 2005)*.*

Previously, ANGPTL4 was expected to bind to the angiopoietin receptor tyrosine kinases Tie1 and Tie2 because of its similarity to angiopoietin proteins. However, it was found that ANGPTL4 does not interact with either Tie receptors, but instead binds to LPL and syndecans as a ligand (Yang et al., 2018). ANGPTL4 consists of a C-terminal fibrinogen-like domain and an N-terminal coiled-coil folding domain. ANGPTL4 (50 kDa) is cleaved within the internal linker region by various proprotein convertases prior to secretion, thus releasing its C-terminal fibrinogen-like domain (38 kDa) and an N-terminal coiled-coil domain (15 kDa) (Figure 1). There may be additional tissue-specific post-translational processing as well, as it has been shown that liver secretes the N-terminal isoform while adipose tissue secretes full-length ANGPTL4 (Yang et al., 2018).

The C-terminal domain is found in circulation as a monomer and the N-terminal domain as an oligomer. The C-terminal fibrinogen-like domain of ANGPTL4 regulates angiogenesis, modulating vascular integrity by disrupting VE-cadherin and claudin-5 (Guo et al., 2014; Okochi-Takada et al., 2014). This indicates that the C-terminus of ANGPTL4 controls vascular permeability and angiogenesis. Previous studies have shown that mice lacking ANGPTL4 displayed impaired angiogenesis and increased vascular leakage during development and in pathological conditions (Perdiguero et al., 2011; Katanasaka et al., 2013), confirming the role of C-terminal ANGPTL4. The N-terminal domain of ANGPTL4 contributes to the LPL-mediated regulation of lipid metabolism (La Paglia et al., 2017) and it is necessary and sufficient to inhibit LPL (Yin et al., 2009; Vienberg et al., 2015). The N-terminal domain binds to LPL, resulting in the conversion of LPL from active dimers to inactive monomers, thereby inhibiting LPL activity (Sukonina et al., 2006).

A previous study found that high-fat fed ANGPTL4 knockout mice showed significantly lower total TG levels than wild-type mice over time (from 4 weeks of age to 24 weeks of age) (Lee et al., 2009). On the contrary, TG levels in both ANGPTL4 transgenic male and female mice were significantly higher than wild-type mice (3-fold and 2.6-fold, respectively) (Köster et al., 2005). From the data published to-date, it appears that ANGPTL4 expression plays a significant role in tissue-specific regulation of LPL activity and is a critical component in the etiology of high-fat diet induced dysregulation of lipid metabolism. What is not known, is the role of ANGPTL4 in satellite cells or undifferentiated myoblasts in response to FFAs. Robciuc et al. demonstrated that ANGPTL4 mRNA and protein levels increased during myoblast differentiation, but there is no data to date on the role of FFA-induced ANGPTL4 in myoblasts or satellite cells (Robciuc et al., 2012).

## The effects of FFAs on ANGPTL4 in skeletal muscle

The activity of ANGPTL4 is regulated by various factors including fasting, chronic calorie restriction, physical activity, high-fat, and high-energy diets (Kersten et al., 2009; Dijk and Kersten, 2014; Van der Kolk et al., 2016; Yang et al., 2018). It is also known that FFAs induce ANGPTL4 expression in skeletal muscle, and that fasting, calorie restriction, and physical activity all induce TG hydrolysis from adipose tissue. Thus, while it is a normal physiological process for ANGPTL4 to be induced following appropriate metabolic signals, herein we are hypothesizing that chronic and excessive fat accumulation in skeletal muscle may produce a pathological response to myogenesis that is mediated, at least in part, by ANGPTL4.

A previous study found that long-chain fatty acids upregulated the expression of ANGPTL4 in human myotubes and that gene expression of ANGPTL4 was significantly increased after treatment with palmitate (16:0), stearate (18:0), palmitoleate (16:1n7), oleate (18:1n9), and linoleate (18:2n6) in primary human myotubes by 10 to 50 fold (Staiger et al., 2009). In addition, similar results were observed with ANGPTL4 protein, with intracellular levels increased with palmitate, oleate, and linoleate treatment in human myotubes. *In vivo*, a meal high in saturated fat increased postprandial ANGPTL4 secretion in obese human forearm muscle (Van der Kolk et al., 2016). While it is clear that FFAs induce ANGPTL4 gene and protein expression in mature skeletal muscle, previous studies have only investigated *differentiated* mature skeletal muscle. It is not known if or how FFAs affect skeletal muscle progenitor cells, and this is an area that should be investigated to fully address the effects of FFAs and ANGPTL4 in skeletal muscle differentiation.

## Effects of ANGPTL4 on molecular signaling pathways involved in skeletal muscle differentiation

**FIGURE 2:**
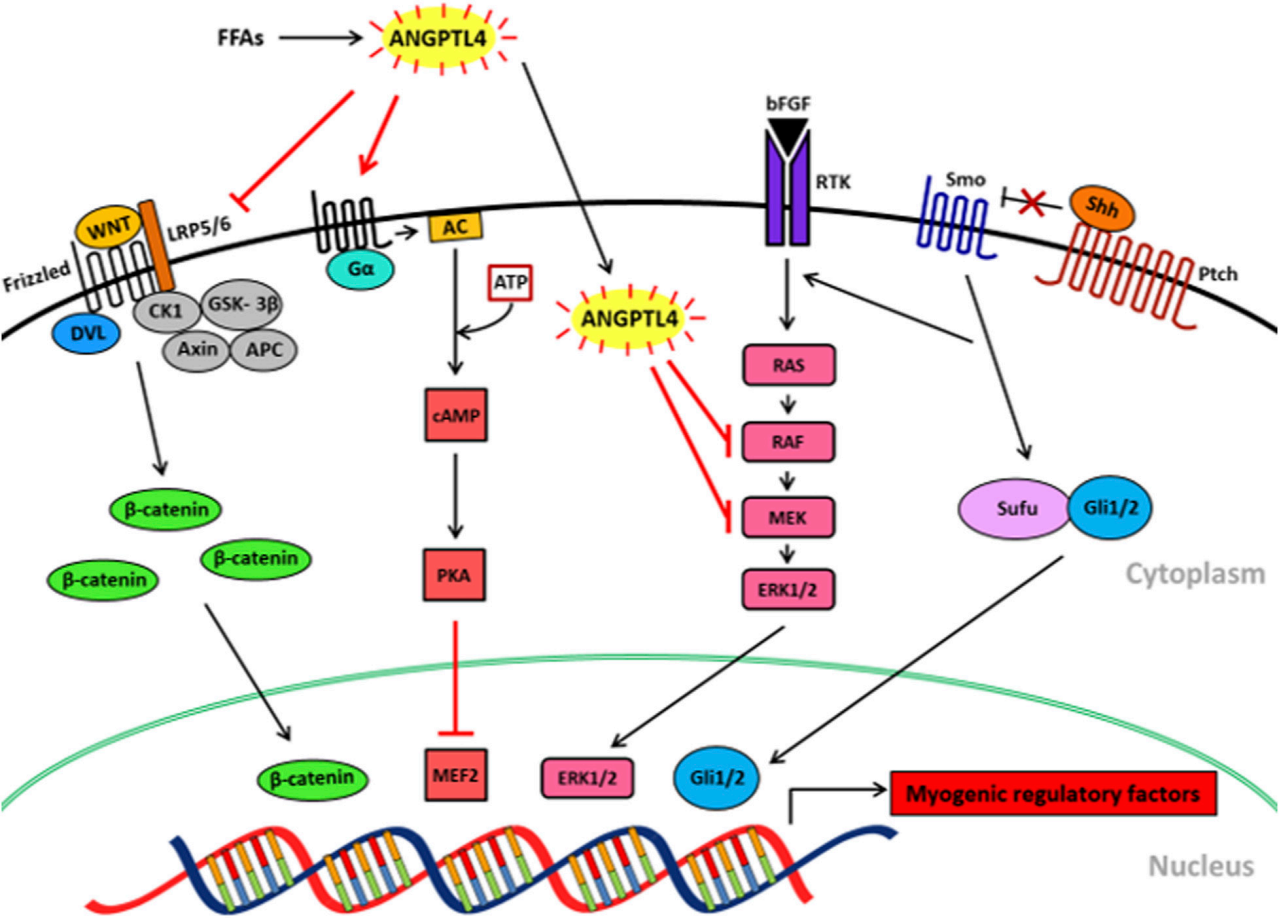
Proposed mechanisms of FFA-induced ANGPTL4 in skeletal muscle differentiation via signaling. FFAs induce ANGPTL4 release in skeletal muscle which downregulates Wnt/β-catenin signaling by inhibiting LRP5/6 receptor binding to Wnt ligands. ANGPTL4 also inhibits bFGF-induced and Shh-induced MAPK/ERK signaling pathways by blocking the phosphorylation of RAF and MEK. ANGPTL4 stimulates cAMP/PKA signaling, resulting in the inactivation of MEF2. Those effects of ANGPTL4 on the signaling pathways may result in the inhibition of skeletal muscle differentiation. FFAs, free fatty acids; ANGPTL4, angiopoietin protein like 4; LRP5/6, low-density lipoprotein receptor-related protein 5/6; DVL, dishevelled; CK1, casein kinase 1; GSK-3β, glycogen synthase kinase -3β; APC, adenomatous polyposis coli; PKA, cAMP-dependent protein kinase; MEF2, myocyte enhancer factor2; AC, adenylate cyclase; bFGF, basic fibroblast growth factor; RTK, a receptor tyrosine kinase; Shh, sonic hedgehog; Ptch, patched; Sufu, surppressor of fused; Gli, glioma-associated oncogene.

Skeletal muscle differentiation is regulated by various signaling pathways such as Wnt/β-catenin, p44/42 MAPK/ERK1/2, Sonic Hedgehog (Shh) and cAMP/PKA signaling pathways (Figure 2). In the following section, we introduce the signaling pathways and explain how they may be affected by diet to regulate skeletal muscle differentiation. Furthermore, we discuss the known and proposed effects of ANGPTL4 on those signaling pathways and their indirect impact on myogenesis.

### ANGPTL4 and Wnt/β-catenin signaling

The Wnt/β-catenin signaling pathway plays an essential role in the regulation of skeletal muscle development and homeostasis (Suzuki et al., 2015b). Wnt ligands belong to a cysteine rich glycoprotein family that is necessary for several biological processes including embryonic induction, cell polarity regulation, and the determination of cell fate (Logan and Nusse, 2004; Kirsch et al., 2017). In the absence of Wnt ligands, destruction complexes are formed by casein kinase 1 (CK1), axin, adenomatous polyposis coli (APC) and the enzyme glycogen synthase kinase-3β (GSK-3β). These destruction complexes phosphorylate β-catenin, resulting in its degradation. Wnt/β-catenin signaling is initiated by extracellular Wnt ligand binding to Frizzled receptors and low-density lipoprotein receptor-related protein 5 (LRP5) and -6. Wnt ligand binding activates intracellular signaling by interacting with Disheveled, a phosphoprotein that facilitates signal transduction. As a result, β-catenin becomes stabilized and accumulates in the cytoplasm. The accumulated active form of β-catenin translocates into the nucleus and functions as a transcriptional coactivator by binding to T-cell factor/lymphoid enhancer factor (TCF/LEF) to induce gene expression (Suzuki et al., 2015a). Consequently, β-catenin activates the expression of MRFs which regulate skeletal muscle differentiation.

Wnt/β-catenin signaling has been shown to facilitate myogenesis by regulating MRFs in both prenatal and postnatal skeletal muscle development leading to the formation of multi-nucleated myotubes (Huraskin et al., 2016). A previous study reported that in the absence of Wnt activity, tissue damage and limited skeletal muscle development occurred, which resulted in death (von Maltzahn et al., 2012). Additionally, β-catenin knockout mice failed to develop skeletal muscle and induced embryonic lethality (Haegel et al., 1995). β-catenin is necessary to form myotomes in somite cells, which is an early stage of myogenesis (Hutcheson et al., 2009). In the absence of Wnt ligands or β-catenin activity, the expression of Myf5 was significantly reduced in mouse embryos, suggesting that Wnt/β-catenin is required for Myf5 activation in somite cells (Ikeya and Takada, 1998; Borello et al., 2006). In addition, the secreted Wnt antagonist frizzled related protein (Frzb1), inhibited embryonic skeletal muscle myogenesis, showing decreased myogenic specification by reducing the expression of differentiation markers including nestin and MHC (Borello et al., 1999). On the contrary, a Wnt3A activating ligand increased myoblast fusion during muscle differentiation by inhibiting GSK-3β and activating β-catenin translocation into the nucleus in C2C12 cells (Pansters et al., 2011).

A recent observation identified that ANGPTL4 downregulates Wnt/β-catenin signaling by inhibiting Wnt co-receptor LPR6 binding to Wnt ligands (Kirsch et al., 2017). The study found that Wnt signaling was significantly improved (approximately 4-fold) in H1703 cells in the absence of ANGPTL4, but not ANGPTL3 or ANGPTL5. In addition, ANGPTL4 knockdown increased the expression of Axin2, a direct Wnt target gene, and β-catenin protein levels in H1703 and HEK293T cells. Increased protein levels of LRP6 were observed in the absence of ANGPTL4, suggesting that ANGPTL4 acts at the Wnt receptor level. Kirsch et al. found that the amino terminal fragment ANGPTL4, but not the carboxyl (C) -terminal, was sufficient to inhibit Wnt/β-catenin signaling with no significant effect from ANGPTL3 and ANGPTL5 peptides (Kirsch et al., 2017).

### ANGPTL4 and MAPK/ERK signaling

In addition to the Wnt/β-catenin pathway, the p44/42 mitogen activated protein kinase/extracellular signal regulated kinase 1/2 (p44/42 MAPK/ERK1/2) pathway is also known to regulate cellular growth and differentiation (Gredinger et al., 1998; Shefer et al., 2001; Sun et al., 2015). It was shown that ERK1/2 activity was elevated during differentiation in C2C12 mouse myoblast cells, showing increased expression of c-*fos,* which is a direct target gene induced by ERK1/2 signaling (Gredinger et al., 1998). Furthermore, ERK1/2 signaling promotes the migration of myoblasts, which is an important step in differentiation by allowing myoblast fusion into myotubes (Dimchev et al., 2013). On the contrary, inhibition of ERK1/2 signaling by an antagonist, UO126, reduced myoblast migration in C2C12 cells. Similar results were observed with another inhibitor of ERK activity, PD098059, which attenuated the fusion of muscle cells by reducing the levels of MyoD (Gredinger et al., 1998).

A previous study found that ANGPTL4 inhibits basic fibroblast growth factor (bFGF)-induced ERK1/2 signaling (Yang et al., 2008). The study reported that the C-terminal domain of ANGPTL4 inhibited bFGF-induced phosphorylation of ERK1/2 but not Akt and p38 MAP kinase. In the presence of ANGPTL4, ERK1/2 activation was decreased by approximately 50% in the HEK293 cells. A mechanism proposed by the study authors is that the C-terminal domain of ANGPTL4 inhibits the activation of RAF-1 and MEK1/2 but not the auto-phosphorylation of FGF receptor and the activation of RAS. RAF-1 is the first target of C-terminal ANGPTL4, followed by MEK1/2, resulting in the inactivation of ERK1/2 MAP kinase. In light of these data, it is suggested that ANGPTL4 peptides (both C- and N-terminal fragments) may play independent roles in inhibition of myogenesis by blocking parallel signaling pathways that regulate skeletal muscle differentiation.

### ANGPTL4 and sonic hedgehog signaling

The morphogenic signaling protein, sonic hedgehog (Shh) is known to promote vascular development and endothelial differentiation in bone marrow mesenchymal stem cells via vascular endothelial growth factor (VEGF) (Rivron et al., 2012; Shi et al., 2018). Shh signaling is initiated when Shh inactivates 12-transmembrane protein Patched-1 (Ptch1) through ligand-binding (Straface et al., 2009). Ptch1 inhibition then releases smoothened (Smo), a G protein-coupled receptor protein, from Ptch1-mediated. As a result, Smo accumulates in the cytoplasm, and induces a downstream signaling series of intracellular reactions which activate the glioma-associated oncogene transcription factors (Gli-1, -2, and -3) by interacting with suppressor of fused (Sufu). The activated Gli proteins are translocated into the nucleus and regulate the transcription of target genes that regulate skeletal muscle cell development (Carballo et al., 2018).

A previous study found that Shh overexpression in bone marrow mesenchymal stem cells significantly increased the protein expression of CD31 and VE-cadherin, increasing angiogenesis in the cells (Shi et al., 2018). The study reported that the activation of Shh increased the expression of ANGPTL4, an angiogenesis associated gene, by approximately 5-fold. In addition, Shh induced the *in vitro* formation of an endothelial capillary network in an artificial tissue, resulting in the formation and regeneration of bone tissue by regulating angiogenic genes such as VEGF and ANGPTL4 (Rivron et al., 2012). It is noteworthy that Shh signaling was impaired in basal cell carcinoma because of the mutation of Ptch1, while mRNA expression of ANGPTL4 was significantly increased (9.77 fold) (Valin et al., 2009). Thus, it is suggested that Shh signaling is associated with ANGPTL4 and their relationship is context-dependent, however, further investigations are required to elucidate precise interaction between Shh signaling and ANGPTL4.

Shh has been found to activate the ERK1/2 pathway to regulate skeletal muscle proliferation and differentiation (Elia et al., 2007; Madhala-Levy et al., 2012). Similar to Wnt signaling, the Shh signaling pathway is involved in skeletal muscle differentiation (Elia et al., 2007) where it promotes the formation of the myogenic lineage in the embryo as well as adult myoblasts and induces myoblast proliferation and differentiation (Elia et al., 2007; Straface et al., 2009; Madhala-Levy et al., 2012). It was found that Shh promotes the terminal differentiation of myogenic cells by expressing MRFs (Madhala-Levy et al., 2012). Shh increased the expression of Pax7, MyoD, and MHC in a dose-dependent manner in myoblasts of adult chickens, (Elia et al., 2007). Furthermore, inhibition of Shh signaling reduced MyoD gene and protein expression (Voronova et al., 2013). In another study, Shh increased MyoG protein levels in C2C12 cells in a dose-dependent manner (Madhala-Levy et al., 2012). Finally, increased Shh activity resulted in increased numbers of myoblasts and nuclei in myotubes in adult skeletal muscle cells (Teixeira et al., 2018). However, whether or not Shh is involved in FFA-induced ANGPTL4 signaling, either directly or indirectly (via Wnt/β-catenin) is unknown or requires further exploration.

### ANGPTL4 and cAMP/PKA signaling

Cyclic adenosine 3′,5′-monophosphate (cAMP) is an intracellular second messenger that is synthesized from adenosine triphosphate (ATP). This process is catalyzed by adenylyl cyclase, which is activated by a stimulatory Gα protein (Sassone-Corsi, 2012). cAMP stimulates a cAMP-dependent protein kinase (PKA), which is a regulatory enzyme in various cellular processes such as cell growth and metabolism, cell division, and DNA replication. Several studies have found that the cAMP/PKA pathway represses myogenic differentiation (Li et al., 1992; Winter et al., 1993; Du et al., 2008). cAMP/PKA signaling pathway inhibits muscle-specific transcription by reducing the activity of MRFs including Myf5, MyoD, MyoG, and Mrf4 (Li et al., 1992). In mouse C2C12 and rat L6 myoblast cells, the activation of cAMP decreased myotube formation, and cAMP almost completely inhibited mRNA expression of MyoG in both cell lines (Winter et al., 1993). cAMP-mediated inhibition of skeletal muscle differentiation occurs through PKA activation, which in turn suppressed the activity of Myf5 and MyoD. Recently, Du et al. found that during skeletal muscle differentiation, the activation of cAMP/PKA signaling pathway decreased MCK promoter activity as well as the transactivation properties of myocyte enhancer factor 2 (MEF2), which activates muscle specific gene expression and thus differentiation (Du et al., 2008). While there are relatively few studies that have examined this relationship, it was shown that cAMP/PKA signaling pathway activation suppresses skeletal muscle differentiation by downregulating the expression of MRFs and inhibiting transcriptional activity of MEF2 (Du et al., 2008).

Interestingly, ANGPTL4 stimulates cAMP-dependent PKA signaling [103, 104]. It was reported that ANGPTL4 is necessary to increase cAMP levels and the PKA-dependent phosphorylation of major components of the downstream signaling (Gray et al., 2012). In addition, ANGPTL4 recombinant protein increased glucocorticoid-induced cAMP levels in mice, increasing TG hydrolysis in adipocyte (Koliwad et al., 2012). Together, it is suspected that ANGPTL4 may inhibit skeletal muscle differentiation by activating cAMP-PKA signaling, however as with the signaling pathways described above, more research into this area is needed.

## ANGPTL4 as a potential mediator of cross talk between FFAs and skeletal muscle differentiation

The link between FFAs and skeletal muscle differentiation remains to be fully elucidated along with a description of potential physiological mechanisms. As previously described, it is clear that FFAs are associated with ANGPTL4 expression in skeletal muscle, and that FFAs are associated with impaired differentiation. It is reasonable to hypothesize that excess long-chain fatty acids that induce ANGPTL4 expression in skeletal muscle cells would mediate suppression of myogenesis by altering intracellular signaling. From the preliminary evidence outlined above, it is likely that FFAs are associated with impaired skeletal muscle differentiation via increased ANGPTL4 expression.

There are several molecular signaling pathways that activate skeletal muscle differentiation including Wnt/β-catenin, Shh, and p44/42 MAPK/ERK1/2. In addition, Shh signaling itself regulates skeletal muscle differentiation, but it also activates ERK1/2 signaling. Unlike those signaling pathways, cAMP/PKA signaling pathway is known to inhibit skeletal muscle differentiation. In light of these findings, several potential mechanisms are proposed through which FFA induced-ANGPTL4 may inhibit skeletal muscle differentiation by suppressing transcription factor expression/activity, Wnt/β-catenin signaling, p44/42 MAPK/ERK1/2 activity and/or Shh signaling, or by activating cAMP/PKA signaling (Figure 2). Future studies will need to be employed to fully examine whether or not any (or all) of these factors play a role in FFA-mediated impairments in myogenesis.

## Summary and future directions

There is a growing body of evidence to suggest that FFAs increase ANGPTL4 expression throughout the course of myogenesis from satellite cell stages through maturation. However, it is not known whether ANGPTL4 is normally expressed in satellite cells or if there is merely a transient response to changes in dietary patterns. Additionally, not all FFAs possess the same biological activity with respect to cell signaling, metabolism, or ANGPTL responses. Therefore, it would be appropriate to assess the impact of various FFAs on satellite cells throughout differentiation to determine if and when ANGPTL4 expression occurs and to what extent it may impact myogenesis. Furthermore, in addition to its well-established role as a mediator of lipoprotein lipase activity, ANGPTL4 exhibits autocrine/paracrine effects and has been shown to suppress the Wnt/β-catenin pathway of differentiation by blocking Wnt binding to its extracellular receptor, low-density lipoprotein receptor-related protein 5/6 (LRP5/6) (Kirsch et al., 2017). Thus, while it is a normal physiological process for ANGPTL4 to be induced following appropriate metabolic signals in mature skeletal muscle, the current review has summarized the impact that chronic and excessive fat accumulation in skeletal muscle may produce a pathological response among resident satellite cells that impairs myogenic potential.

It is important for any potential link between dietary FFAs and skeletal muscle differentiation to be fully elucidated, along with a description of potential physiological mechanisms. As previously described, it is clear that 1) FFAs are associated with ANGPTL4 expression in skeletal muscle (Staiger et al., 2009; Son and Paton, 2020), 2) that ANGPTL4 reduces Wnt signaling (Kirsch et al., 2017), and 3) that reduced Wnt activity leads to impaired skeletal muscle differentiation (Polesskaya et al., 2003; von Maltzahn et al., 2012; Abraham, 2016). Therefore, it is reasonable to hypothesize that excess long-chain FFAs that induce ANGPTL4 expression in skeletal muscle cells, would mediate suppression of myogenesis through a reduction in Wnt signaling. From the published evidence outlined above, it is likely that FFAs are associated with impaired skeletal muscle differentiation via increased ANGPTL4 expression. Future studies should be designed to test whether ANGPTL4 acts as a mediator of cross talk between dietary FFAs and impaired skeletal muscle differentiation via reduced Wnt/β-catenin signaling in satellite cells.
